# P-245. Not in the Bundle: Mitigating Central Line-Associated Bloodstream Infections in the Trauma ICU through Central Line Access Restriction

**DOI:** 10.1093/ofid/ofae631.449

**Published:** 2025-01-29

**Authors:** Thomas Pustorino, Marina Keller, Jamie Palazzo, Charmaine Mikaya, Moira Quinn, Phyllis Yezzo, Kartik Prabhakaran, Donald S Chen, Stephanie Kennedy, Chinwe Anekwe

**Affiliations:** Westchester Medical Center, Rye, New York; Westchester Medical Center, Rye, New York; Westchester Medical Center, Rye, New York; Westchester Medical Center, Rye, New York; Westchester Medical Center, Rye, New York; Westchester Medical Center, Rye, New York; Westchester Medical Center, Rye, New York; Westchester Medical Center, Rye, New York; Westchester Medical Center, Rye, New York; Westchester Medical Center, Rye, New York

## Abstract

**Background:**

Central line-associated blood stream infections (CLABSI) pose a significant threat in healthcare. The CDC CLABSI reduction bundle focuses primarily on optimizing line insertion and has few recommendations addressing line maintenance. This ignores most of the line’s lifespan and risk for infection.

**Figure:**
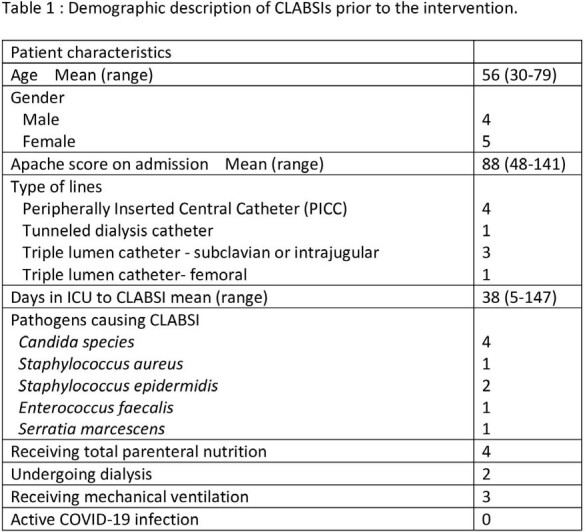

**Methods:**

The single center observation study sought to reduce risk for CLABSI by focusing on central line maintenance and access within a Trauma Intensive Care Unit (TICU). Nurse epidemiologists observed front line staff and noted that many central lines had a long dwell time and were accessed multiple times a day. In order to reduce the number of line accesses, a process was instituted where blood draws were preferentially obtained via peripheral blood draw or arterial line. Education regarding line maintenance and proper line access technique was reviewed and reinforced. Compliance with this process was prospectively monitored.

**Figure:**
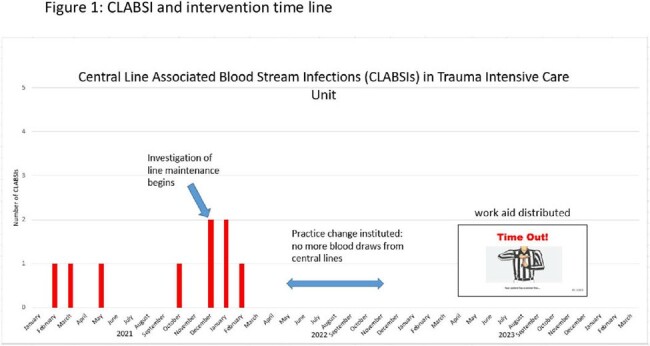

**Results:**

From February 2021 to February 2022, the TICU experienced a surge of 9 CLABSIs with all the lines having an extended dwell time of 5-147 days (mean 69 days). Of these patients, 4 were receiving total parental nutrition and 4 were undergoing dialysis (Table 1). Over the summer of 2022, education was used to change practice in the TICU and stop bloodwork drawing from central lines. After the process change, no CLABSIs were reported for over 24 months, until March 2024 (Figure 1). To sustain progress, a line access audit and blood draw audit were developed to ensure vigilant hand hygiene and scrub-the-hub technique were implemented and to ensure that blood draws were not obtained from central lines. As of March 2024, >95% of blood draws were not obtained from central lines decreasing line access. Job aid cards were developed to teach and reinforce how to critically evaluate blood draws (Figure 2).

**Figure:**
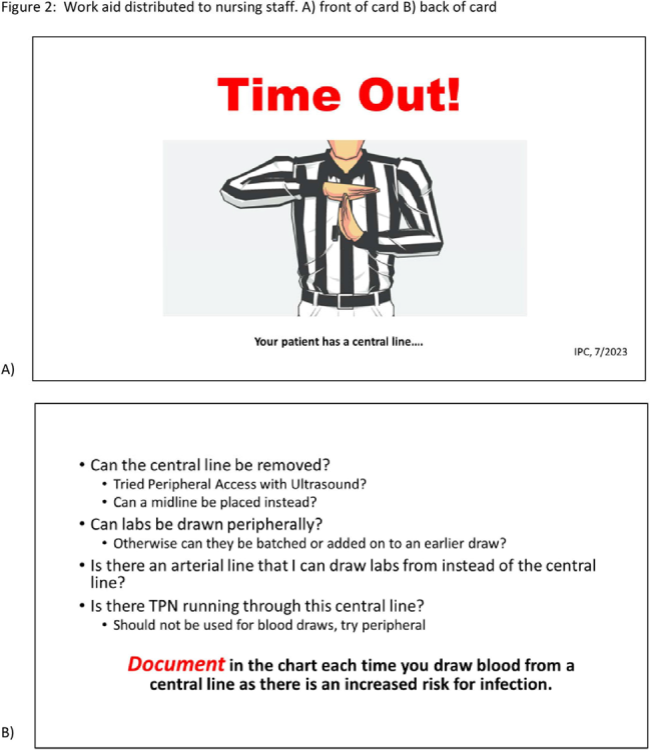

**Conclusion:**

The study underscores the likely connection between blood draw practices and risk for line contamination. Minimization of central line access can decrease the chance of line contamination; avoiding blood draws are an effective way to radically reduce line access and bring CLABSI rates to zero.

**Disclosures:**

**All Authors**: No reported disclosures

